# Experiences of Tobacco Use among Chinese Individuals with Schizophrenia in Community-Based Residential Settings: A Qualitative Study

**DOI:** 10.3390/ijerph17010321

**Published:** 2020-01-02

**Authors:** Yim Wah Mak, Vico C. L. Chiang, Alice Yuen Loke

**Affiliations:** School of Nursing, The Hong Kong Polytechnic University, Hung Hom, Kowloon, Hong Kong, China; vico.chiang@polyu.edu.hk (V.C.L.C.); alice.yuen.loke@polyu.edu.hk (A.Y.L.)

**Keywords:** tobacco use, smoking cessation, schizophrenia, qualitative study

## Abstract

Although there is a high prevalence of smoking among individuals with schizophrenia, no previous attempt has been made to explore experiences of tobacco use and cessation within a Chinese sample of this population. A qualitative descriptive study was conducted to explore through the use of individual and semi-structured interviews the experiences of tobacco use and quitting in a sample Chinese population with schizophrenia. Twenty-three eligible participants with schizophrenia who currently smoke were recruited from three community residential mental health service settings. Four main themes representing the experiences of the participants were uncovered in this study: (1) smoking and illness; (2) environment and culture; (3) reasons for smoking; and (4) beliefs about cessation methods. The findings indicated that the participants considered the disease to be far more harmful than smoking, and perceived many benefits to smoking. Although some thought of quitting at times, most gave up the idea or failed to quit due to internal factors such as disease-related suffering, the use of an avoidance coping strategy, and a lack of cessation support. The most notable finding concerns the use of avoidance coping by the participants, who relied on smoking as a way to avoid the suffering brought about by the disease.

## 1. Introduction

People with schizophrenia have about a 20% lower life expectancy than the general population [1,2] Smoking is believed to account for the majority of the excess mortality among individuals with schizophrenia [3,4,5,6,7], with such smokers being at a twelve times greater risk of dying from cardiac-related illnesses than non-smokers with schizophrenia [8]. In many countries, individuals with schizophrenia also have the highest prevalence of smoking, a higher dependence on nicotine, and a significantly lower smoking cessation rate than is the case among the general population and among smokers with other psychiatric illnesses [9,10,11,12,13]. Various studies have been conducted exploring the reasons for the high prevalence of smoking among people with schizophrenia. The view is that there are complex interactions between the intake of nicotine and neurobiological [14,15,16,17,18], psychosocial, and behavioral factors [19,20,21]—for example, the effects of the release of dopamine in the brain, coping, managing stress, and so on.

There have been very few in-depth studies on tobacco use and cessation from the perspective of psychiatric populations [22,23,24,25]. Among them, only one study has explored beliefs and attitudes regarding the smoking behaviors of people with schizophrenia [22]. The study found that people with chronic schizophrenia believed that smoking could help to relieve their anxiety and negative symptoms, and therefore were not thinking of quitting smoking. Factors that might influence smoking and quitting from the perspective of Chinese people with schizophrenia have also been relatively under-examined. About one-third of the world’s smokers are in China and a direct understanding of their experiences is needed [26]. In Hong Kong, the Government has adopted several tobacco-control strategies to safeguard public health, comprising legislation, tax measures, and public education on a smoke-free life. In addition, the Government and NGOs operate a quit-line and district and hospital-based smoking cessation services. However, those services are not particularly targeted at people with mental health illnesses [27]. Little is known about the experiences and personal views of people with schizophrenia on the role that tobacco plays in their lives. This lack of knowledge may lead to failures in the design and implementation of effective smoking cessation programs targeting the specific needs of this population. This is a report on a qualitative study of the experiences of a group of Chinese patients with schizophrenia in using tobacco and quitting smoking. 

## 2. Methods 

### 2.1. Design

A qualitative descriptive design was adopted in this study for an in-depth exploration of the experiences and perceptions of schizophrenic patients [28], in an approach where the researchers would not manipulate the findings or impose any *a priori* ideation [29]. A qualitative descriptive design was chosen because it is appropriate for examining the smoking and quitting experiences of people with schizophrenia who have received little attention in the literature [28].

### 2.2. Sampling and Recruitment

The participants were recruited from community residential mental health service settings, including two hostels and a half-way house of a non-governmental organization (NGO), all of which provide counseling and transitional residential care. The purpose of the study was explained to the in-charge of the target institutions. Permission was obtained from the institutional in-charge to recruit subjects at the institutions. Convenience sampling was used for this study, whereby case managers of the participating NGO identified potential participants for the study. The criteria for inclusion were individual residents who (1) had received a diagnosis of schizophrenia; (2) had been referred to the settings by medical doctors; (3) were currently using tobacco; (4) had expressed an interest in taking part in a face-to-face individual interview; and (5) were able to communicate in Cantonese. Those with disorientation, developmental disabilities, and/or organic conditions were excluded from the study. 

The potential participants were told the purpose of the study and screened for eligibility by the first author and her students. Arrangements were made to interview the participants at times convenient to the participants and at the centers where they received services. Those potential participants who were eligible were invited to join this study, and the written informed consent of those who agreed to take part in this study was obtained. In addition, guardians of these participants provided consent on their behalf. All of the procedures performed in this study were in accordance with the ethical standard of the 1964 Helsinki declaration and its later amendments. Ethical approval was also obtained from the Human Subjects Ethics Committee of the Hong Kong Polytechnic University (HMRF10111861) and written consent was obtained from all participants.

### 2.3. Data Collection

Data were collected by the first author and six post-graduate nursing students who were all trained to conduct semi-structured interviews. The first author served as the primary interviewer for this study, while the six students took notes of the interviews. A set of guided, open-ended questions (Table 1) was used to obtain rich narrative data. Interviews began with general questions about the participants’ current smoking habits and smoking history, along with their experiences of attempts to quit smoking. The participants were then asked whether they saw a connection between smoking and their mental illness. Their perceptions and experiences with smoking or cessation, barriers to cessation, and views on cessation methods were also explored. 

Demographic data including information on age, gender, and socioeconomic status were obtained. Each interview took approximately thirty to forty-five minutes and was audio-recorded. The interviews were conducted in Cantonese. Following the interview, the audio-taped data were transcribed verbatim and analyzed. Essential findings were then translated into English for reporting by the first author, who is proficient in both English and Cantonese. The translation was then checked by a native English-speaking technical writer. 

### 2.4. Data Analysis

The interview data were examined and analyzed through latent qualitative content analysis, which is based on an inductive approach [30]. The goal of such an analysis was to identify the main categories (themes) in the data, and patterns among the sub-categories [31]. The unit of analysis was a statement from the interview transcripts. The analysis facilitated the task of exploring and interpreting the underlying meanings of the texts (data), which led to the emergence of meaningful units of sub-categories. A name was given to each sub-category corresponding to the meanings of its coding. Finally, the sub-categories were condensed to achieve the status of a theme [30]. 

To ensure the trustworthiness of the results, each transcript was analyzed independently by two investigators. They then met to discuss the data and come to a consensus on the themes. The researchers analyzed the data until they reached the point of data saturation when no new themes emerged. In selecting quotes for reporting we took into account a number of criteria. As far as possible, we hoped to include at least one quote from each of the 23 participants and to select the clearest quotes for reporting. In the end, quotes from 20 participants were selected for reporting in this manuscript. 

## 3. Results

### 3.1. Sample Characteristics

The 23 participants ranged in age from 26 to 62 (m = 49.13, SD = 7.79). Only one was female. All of the participants were living with roommates in the residential facilities of the three NGOs. Prior to being institutionalized, the majority lived in public housing provided by the Hong Kong government.

Most of the participants had a secondary level of education, had been admitted to a psychiatric hospital (78.5%), and had received regular psychiatric follow-ups in the last year (93.1%). Almost all of the participants had started smoking on or before the age of 25 (~91%). The majority (>75%) had smoked for at least 20 years. One third had previously attempted to quit smoking. Table 2 describes the summarized profile of the 23 participants (P1–P23).

### 3.2. Themes

Four main themes emerged from the analysis, (1) smoking and illness; (2) environment and culture; (3) reasons for smoking; and (4) beliefs about cessation methods.

### 3.3. Smoking and Illness

Two sub-themes emerged during a further analysis of this theme: (1) the perceived relationship between smoking and illness; and (2) the perceived relationship between smoking and medications. From the data analysis, we see that the participants had different and contradictory views and experiences relating to their smoking and schizophrenia. 

#### 3.3.1. Perceived Relationship between Smoking and Illness

When the participants were asked what they thought was the relationship between schizophrenia and smoking, most said that there was no relationship. For example, P7 commented that, *“It has nothing to do with smoking. There are many people who don’t have mental illness … a lot of people smoke but have no mental illness at all.”*

Although the participants saw no relationship between their illness and smoking, they were generally not too serious about quitting smoking. They perceived quitting to be very challenging, with some relating the difficulties to being a patient with schizophrenia. For example, P21 described having schizophrenia as already tough enough and said that he could not quit smoking or it might intensify his psychotic symptoms. He said, *“Being schizophrenic involves too much suffering, I can’t quit. … If I could recover from schizophrenia after stopping smoking, I would do that … but I wouldn’t recover from schizophrenia. I can’t quit … even if I did I would smoke again.”* The interviewer asked P21, “Why is that so?” P21 explained that, *“I hear voices (if I quit). I hear the TV scolding me, the radio also scolds me, people around and the entire world scold me too.”*

#### 3.3.2. The Perceived Relationship between Smoking and Medications

Several participants linked smoking with the taking of antipsychotics. 

For example, P23 claimed that antipsychotics increased his urge to smoke: *“I really need to take one to two puffs of a cigarette after taking the antipsychotic medications … maybe because those medications make me crave a cigarette.”* P4 reported that he smoked more when he suffered from more side-effects after taking antipsychotics, *“If the side-effects of the antipsychotics are strong, then I will take more cigarettes. … If the side-effects are not that strong, I can then take fewer cigarettes.”* When P12 was asked about the effects of smoking in relation to medications, he replied that smoking helped to reduce the side-effects of antipsychotics: *“It helps … I don’t feel that tired, and that sleepy. Sometimes after the injection, I feel sleepy and very tired. … When I smoke, it stimulates me to feel energetic, and I do not worry about being sleepy.”*

### 3.4. Environment and Culture 

Two sub-themes were discovered during a further analysis of this theme: (1) cigarette intake depends on institutional regulations on smoking; and (2) the institutional environment affects cigarette intake. In essence, since some settings allow smoking while some do not, it was clear that schizophrenic patients who smoke and live in a residential care setting will or will not smoke depending on the smoking regulations and culture of that institution.

#### 3.4.1. Cigarette Intake Depends on Institutional Regulations on Smoking

Many of the participants in this study reported that their cigarette intake depended on the smoking regulations of the institutions. P4 refrained from smoking when the environment did not permit smoking. P4: *“Hmm … we’re not allowed to smoke in the ward (in the hospital) and so we just don’t smoke.”* Participants, like P17, who had been admitted to hospital, also reported that they restricted their cigarette intake due to strict monitoring by hospital staff. P17 stated: *“Our hospital has very strict monitoring … if you smoke; they will put you in a correctional room.”*

Some participants reported that they stopped smoking while they were in hospital due to the smoke-free policies there. However, like P15, most resumed smoking after being discharged. For example, P15 stated, *“(I) did not smoke for three months in the hospital because of the restrictions. I smoked again after I was discharged.”*

On the other hand, a few participants reported that they smoked in the hospital when they had access to cigarettes. one participant, p17, reported that he smoked in hospital as his *“family members brought cigarettes to the hospital.”* another participant, p20, mentioned that he had smoked in the hospital because someone sold cigarettes in the hospital where he was staying. 

#### 3.4.2. The Institutional Environment Affects Cigarette Intake

Another issue frequently raised by the participants was that many of their peers smoke in the community-based residential settings to which they had moved, and this environment had influenced them to resume smoking, even though some had already quit following hospitalization. One of the participants, P5, stated, *“In my previous dormitory (of the community-based residential setting), more than 70% of people smoked... everyone smoked, and very naturally you will smoke too.”*

Two participants, P5 and P12, mentioned that the mundane environment in their community-based residential area or day care center had caused them to start smoking *“The previous hostels where I lived … I always stayed there… with nothing to do there. Then I always smoked. I applied for a day-care hospital, joining some groups … making artwork. … Then I would smoke less frequently.”*

However, many, including P9, tried to find ways to smoke without violating the smoke-free regulations, such as by smoking somewhere else during work or nearby but outside of their residential block, to ensure that they did not violate the rules concerning smoking “inside” the residential area. For example, P9 reported that, *“When the manager is back, we will have to go near the rubbish bin near the entrance (of the community-based residential area) to smoke.”*

### 3.5. Reasons for Smoking

Four sub-themes emerged during a further analysis of this theme: (1) moods or feelings leading to a desire to smoke; (2) disregarding the harmful consequences of smoking; (3) convincing oneself of the positive effects of smoking; and (4) justifying smoking as a norm.

#### 3.5.1. Moods or Feelings Leading to a Desire to Smoke

The majority of the participants described smoking as having positive effects, focusing particularly on how smoking helped them to cope with or escape from negative emotions or moods. They also talked about the different moods that contributed to their tendency to smoke. For example, moods such as boredom were reoccurring emotions that triggered a desire to smoke (e.g., P10 said, *“Being stuck in a day care center … I felt so pathetic and bored … and I started smoking again”*). Many participants said that smoking was a way of coping with their routine and with the structured institutional life (e.g., according to P15, smoking helps *“to kill time easier... it gives me something to do.”* A number of participants believed that cigarettes helped to relieve everyday stresses, such as workplace pressures (e.g., P3 said, *“Sometimes when I work … I face some job stress … therefore, I want to smoke”*) and negative emotions (e.g., P11 noted, *“When I am upset and feel troubled, I will smoke”*). Many strongly expressed the view that smoking acts as a psychological support for them. To some, smoking can be as simple as just satisfying their craving to smoke. 

To other participants, smoking brought a sense of comfort or enjoyment (e.g., according to P15 *“After two hours of work, I want to take some rest; I will smoke as a way to enjoy myself”*). Some believed cigarettes enhanced their alertness and concentration (e.g., P9 reported, *“I rely on cigarettes when I work. … After I smoke … I can then concentrate... and work efficiently”*), while others believed that cigarettes provided them with companionship over the years and throughout their journey of mental illness (e.g., P11 said, *“The meaning of smoking is that it has accompanied me for many years. … At that time when I was just 19 years old and started smoking, that thing (schizophrenia) happened … smoking has accompanied me for many years”*). In addition, smoking serves a way to engage with people or to make friends (e.g., P16 said, *“The attractive thing about smoking is … being able to make more friends”*). It is clear that smoking serves a special purpose for the majority of the participants.

#### 3.5.2. Disregarding the Harmful Consequences of Smoking

Many participants were able to describe the harmful effects of smoking on physical health, but some did not think that they themselves would suffer from those harmful consequences. When asked whether they were afraid of developing cancer due to smoking, P22 replied, *“I am not scared … it won’t happen to me that easily,” while* P11 noted “*I have been smoking for more than 20 years; there has been no problem. Then what problem would there be?*” Some of the participants believed that herbal tea or beer has a detoxifying or soothing effect on the system, so that drinking tea or beer can reduce the harmful effects of smoking on health. 

#### 3.5.3. Convincing Oneself of the Positive Effects of Smoking

Furthermore, some participants reported that when faced with the dilemma of whether or not to smoke; they often perceived the positive effects of smoking to be greater than the negative effects. Smoking helped them to deal with boredom, stress, and emotional upsets, and provided them with comfort and companionship. For example, P17 described how he struggled over the issue of smoking, before finally deciding to give up quitting after weighing the pros and cons. 

Most participants generally did not think too much about smoking or quitting, as they had come to regard smoking as a kind of *“habit”* and had *“gotten used to smoking.”* For example, P3 described smoking as part of his daily routine, *“[Smoking]... is like … a part of life, similar to eating.”*

#### 3.5.4. Justifying Smoking as a Norm

When asked about feelings and experiences relating to their smoking behaviors, some participants stated that smoking is very common among their peers (e.g., according to P11, *“[Smoking] does not disturb me at all—I have a lot of friends and relatives who smoke”*). Some justified their smoking behaviors by referring to significant figures (e.g., P21 said, “*Deng Xiaoping was a previous leader. He smoked and therefore, every one of us can smoke. … He also smoked in front of the television cameras. … Even Deng Xiaoping could smoke, therefore, I smoke as well”).* Some compared smoking with other addictive behaviors such as taking drugs, gambling, and drinking coffee, and thought that smoking was a relatively healthier habit. For example, as P13 put it, *“Some people gamble, I don’t gamble, neither am I addicted to drinking coffee. I only have nicotine cravings. If I smoke, then I won’t have diabetes … drinking coffee is also not good for one’s health.”*

Factors contributing to the cessation decision and successful quitting.

Two sub-themes were discovered during a further analysis of this theme: (1) the influence of significant others contributes to the cessation decision; and (2) quitting relies on self-control. Some participants claimed that they would not want to participate in smoking cessation programs even if such programs were available, as they perceived them to be ineffective even though they had not tried them. These participants repeatedly said that whether or not one smokes depend on oneself, in particular one’s willpower and self-control. For example, P12 summed up the belief of many participants that self-control was what it took to quit smoking or reduce their cigarette intake.

#### 3.5.5. The Influence of Significant Others Contributes to the Cessation Decision 

The participants often mentioned the influence of significant others, including family members, friends, and peers, on their decision to quit smoking. Familial influence was particularly important. P1 hinted that his family condemned his smoking; as a result, he hides from his family his habit of continuing to smoke. P6 mentioned that she cut down on the number of cigarettes that she consumed because of her mother’s encouragement.

*P1: Errr … the difficulty is, my sister was studying in university, she believes in God so she persuaded me to quit. But I felt disappointed in myself. … Every time I went to church I would sneak into the toilet and smoke secretly, but my sister said, “Don’t do it—your going to church is your own business.” I really didn’t listen, but I tried to restrain it (smoking needs), but I couldn’t bear it anymore. I went to another church for the service, but I couldn’t stop myself again, I went to the toilet to smoke again. Oh, not the toilet—outside, outside of the church.*

#### 3.5.6. Quitting Relies on Self-Control

The participants regarded smoking cessation programs or replacements for smoking as not useful in helping them to quit smoking. For example, P21 described his experiences with nicotine chewing gum and nicotine patches as being useless in helping him to reduce his cigarette intake.

*Interviewer: You tried (nicotine) chewing gum before—did you feel that it was effective (in helping you to quit smoking)?*

*P21: It was just chewing gum … it was not useful. … I always apply (the patch), but nothing has changed.*

## 4. Discussion 

Overall, our findings indicated that smoking played an important role in the lives of individuals with schizophrenia. Although they were ambivalent about smoking and quitting at times, the feeling was not strong enough to prompt many of them to quit, due to the lack of cessation support that they received throughout their journey of recovering from mental illness. The findings of the present study also provided important clues on how the participants used smoking as a source of comfort and a form of avoidance coping.

Most participants did not perceive smoking to be related to schizophrenia. However, some who suggested that smoking may have unique functions for individuals with schizophrenia typically saw those functions as being related to reducing the side-effects of antipsychotics, a finding consistent with that from a previous study [32]. Similar to a previous study on people with chronic schizophrenia, some participants believed that smoking benefited them by reducing their anxiety and negative psychiatric symptoms [22]. Furthermore, similar to some previous studies [20,23,33], we found that the participants believed that quitting might intensify their psychotic symptoms, suggesting a self-medication model [34]. We also found that participants believed that smoking might reduce the efficacy of medications, and thus have a negative effect on the prognosis of the illness. This view has received some support from a review of studies indicating that smoking reduces the speed at which some antipsychotic medications are metabolized; thus, smokers with schizophrenia may require higher doses of medications to control their symptoms [35]. In addition, a few participants believed that having schizophrenia makes quitting smoking more challenging. This was in line with previous research showing that individuals with schizophrenia encountered great difficulties in quitting [9]. Future research is needed to explore in greater depth how living with schizophrenia makes quitting smoking more difficult. It may be important for clinicians to address this issue when devising smoking cessation treatments.

With regard to their attitudes towards smoking and quitting, these were mostly similar to those reported in previous studies for the general population [36] and for individuals with schizophrenia [21,23,24,37,38]. Our participants generally lacked the motivation to quit smoking and were not considering changing their behaviors in this area. The participants in this study avoided the issue of their susceptibility to the harmful health outcomes of smoking and provided justifications for their smoking behaviors. They also found ways to minimize the negative effects of smoking, such as by using illicit cigarettes to reduce the level of nicotine that they inhaled. All of this suggests that they had a tendency to utilize avoidance-oriented coping strategies including minimizing or denying the seriousness of the situation, modifying or eliminating the conditions that gave rise to the problem, and changing their perceptions of an experience in such a way as to neutralize the problem. This is consistent with the findings of a previous study showing that avoidance-oriented coping is common among individuals with schizophrenia [39]. Psycho-therapy such as Acceptance and Commitment Therapy (ACT) might be useful for this population, as ACT teaches participants acceptance-related skills in order to reduce avoidance and increase psychological flexibility [40]. It is believed that through these strategies, the participants will be able to re-contextualize problematic cognitions and have a more realistic view of the negative impact on them of smoking, which in turn may motivate them to reduce their cigarette intake. 

Furthermore, our findings suggest that the motivation to start quitting smoking was not internalized by the smoke-free policies and culture in mental health institutions. Most of the participants reported that they smoked if the environment allowed them to do so or if they had access to cigarettes. However, many of the participants reported that, despite quitting, they resumed smoking upon being discharged from the hospital, when no longer subject to restrictions or due to peer influence in residential settings. Similarly, a review concluded that no long-term untoward effects were observed following the introduction of a ban on smoking among psychiatric patients [41]. From the findings of this study, we understand that the key to prompting individuals with schizophrenia to contemplate quitting smoking or motivating them to take action to quit smoking does not lie in measures such as restricting smoking to specific areas because participants would resume smoking once they moved to facilities that do not restrict smoking. Instead, the solution seems to lie in whether someone is able to walk with these individuals with schizophrenia and be a constant presence with them in their life journey (i.e., be their companion), which may eventually give them the strength to quit.

In addition, our findings indicated that the participants generally had little knowledge of current smoking cessation methods, or harbored misunderstandings about these methods. It seems that throughout the trajectory of their recovery from mental illness, the issue of smoking had been rarely addressed and there was a lack of education regarding smoking cessation strategies. Indeed, this point was echoed by the participants, who said that no cessation programs were available to them and no staff members in the community-based residential area promoted smoking cessation. It is essential to train mental health professionals to take an active role in addressing smoking as part of mental health treatment or routine clinical care, as well as to encourage them to view smoking cessation as a priority or responsibility. This may include assessing the patients’ tobacco use, providing cessation counseling, or referring the patients to local resources for additional information and cessation support. 

When asked about their thoughts on current smoking cessation programs, the majority of participants thought that they were ineffective, whether or not they had used these programs before. On the one hand they believed that quitting depends on one’s willpower and self-control (internal factors); on the other hand, they felt that they would only be able to quit if smoking were banned, suggesting that they have encountered internal barriers to changing their behavior. This has implications for current smoking cessation programs. Effective smoking cessation interventions have to address the patients’ internal barriers to change, and understand and address their psychological need to smoke.

### Limitations

One limitation of this study is that the findings were based on the views of a convenience sample of Chinese participants living in a community-based residential setting. Therefore, the findings of the study may not be generalizable to Chinese individuals living in other settings, to other ethnic groups, or to the population of people with schizophrenia in general. Another limitation of this study is that, on average, the participants had been 27 years of age at the onset of their illness, and most of them had smoked before that. Recall bias could not be totally eliminated. The findings of the study cannot be generalized to Chinese individuals whose illness has been of a shorter duration, as they may have had very different experiences related to their illness and smoking. Moreover, patients with schizophrenia are a heterogeneous group, and 23 participants may not be a large enough sample to come to a firm conclusion about the experiences of such a group. Nevertheless, the use of a qualitative methodology in this study allowed for rich data to be gathered on factors that influence smoking and quitting from a sample of patients with schizophrenia, which could not have been obtained using aggregated quantitative measures.

## 5. Conclusions

Overall, the findings indicate that individuals with schizophrenia face many barriers to quitting smoking, including internal factors (i.e., psychological needs, illness-related difficulties, a tendency to utilize an avoidance coping strategy, and a lack of willpower and self-control) and external factors (i.e., peer influence in community-based residential settings, the lack of available cessation programs, and limited cessation support within mental health services).

Possible useful strategies to pursue in the future include: (1) encouraging clinicians to address the internal barriers to quitting and the psychological needs of individuals with schizophrenia; (2) enforcing a smoke-free policy within hospitals and in other outpatient mental health institutions including community-based residential settings; (3) helping individuals to develop the skills to increase their acceptance of the psychological difficulties of quitting smoking; and (4) providing people with schizophrenia with the necessary support to access smoking cessation services. These measures may address some of the barriers to quitting faced by people with schizophrenia, which in turn will reduce the prevalence of smoking among this group.

## Figures and Tables

**Table 1:** Interview Guiding Questions.

Can you me when and how you or others discovered that you have a mental health condition? How do you feel about your current mental health condition? How do you feel about smoking? How do you feel about the saying that using tobacco may be related to mental illnesses? Please share with me your experiences of smoking in your daily life. How do you view this/se habit/s in relation to your mental health situation? (Prompt): To what extent does/do this/se habit(s) and your mental illness shape your daily habits and life, for example, your daily routine, your family relationships, your self-management abilities, finances, and career? When you are feeling bad or confused, how would you usually deal with this? Would smoking help you to deal with your feelings and thoughts? And how? What do you think could ultimately help you to deal with your mental health condition? And how? Have you ever thought of quitting smoking? If yes, what did you do and how do you feel about it? What forms of health care support in the community have you found to be of help in dealing with your mental health condition and smoking? What forms of health care support do you think are lacking in the community?

**Table 2:** Characteristics of the participants’ smoking behaviors (N = 23).

Characteristics	Range	Mean	SD
Years of smoking	8–37.5	27.66	8.35
Age of smoking	12–35	19.96	5.14
Daily cigarette consumption	3–45	17.14	9.89
